# Self-efficacy and Use of Digital Health Care and Social Welfare Services Among Incarcerated People: Cross-sectional Survey Study

**DOI:** 10.2196/36799

**Published:** 2022-05-31

**Authors:** Teemu Rantanen, Eeva Järveläinen, Teppo Leppälahti

**Affiliations:** 1 Laurea University of Applied Sciences Vantaa Finland

**Keywords:** digital inclusion, digital exclusion, digital divide, digitalization in prisons, incarcerated people

## Abstract

**Background:**

The digitization of health care and social welfare services creates many opportunities for the rehabilitation of incarcerated people and their preparation for release from prison. A range of digital platforms and technology solutions have been developed that offer multiple opportunities to handle private matters either by video conference, email, or some other digital format during imprisonment. However, incarcerated people have limited access to digital health care and social welfare services, and face challenges related to shortcomings in their digital skills and self-efficacy.

**Objective:**

This article assessed the significance of incarcerated people’s self-efficacy in terms of their sense of control over the use of digital health care and social welfare services.

**Methods:**

A cross-sectional study was conducted using a questionnaire. Research data were collected from 11 prisons in different parts of Finland, and a total of 225 incarcerated people responded to the survey. Statistical analyses were conducted using the Pearson product-moment correlation coefficient, 2-tailed t test, linear regression analysis, and Hayes bootstrapping method.

**Results:**

The results showed the significance of both general and internet-specific self-efficacy, which appear to be more important for the use of digital health care and social welfare services than factors related to a person’s socioeconomic background or sentence. Age was negatively correlated with perceived control over the use of digital health care and social welfare services. Furthermore, the study emphasized the importance of support from family and friends, as well as prison employees.

**Conclusions:**

The digitalization of prisons offers many opportunities, but special consideration should be given to how the digitization of health care and social welfare services responds to the needs of incarcerated people in terms of their integration into society and the prevention of recidivism. During imprisonment, attention should be paid to strengthening the digital skills of incarcerated people, with support provided by prison employees. In addition to providing guidance on the use of individual digital services, the study recommends strengthening the general digital skills of incarcerated people, as well as developing their life management skills.

## Introduction

### Role of Digital Health Care and Social Welfare Services in Supporting a Desistance From Crime

Recent discussions on the rehabilitation of incarcerated people and their re-entry into society have emphasized the perspective of a desistance from crime [1-4]. Breaking away from crime is often a very challenging and long-lasting social process for a person with a history of crime, which can include single sudden events [5] or a series of events in a person’s life [2,3]. It is crucial to ensure that timely and adequate support and rehabilitation services are made available during the prison term and in the release phase, and that support continues to outside life [4,6,7]. In addition, while addressing the prevention of recidivism, attention should be paid to the social situation and its improvement [6,8].

Previous studies have argued that incarcerated people should have access to digital services already during their imprisonment, in order to facilitate their integration into society [7,9]. Consistent with this, a range of digital platforms and technology solutions have been developed that offer opportunities to handle private matters either by video conference, email, or some other digital format during imprisonment, with the aim of supporting incarcerated people’s re-entry into society [10-12]. Digitalization in prison makes it possible for incarcerated people to use digital health care and social welfare services, participate in distance learning or rehabilitation, and communicate with family or different authorities [13]. Reisdorf and Rikard [7] have highlighted that both offline and online rehabilitation measures should be targeted during imprisonment and in the re-entry phase, in order to strengthen returnee agency in a digital society. Furthermore, Ogbonnaya-Ogburu et al [14] emphasized the importance of digital literacy in relation to job-searching skills, which also makes it easier for incarcerated people to re-enter society.

Digital and social inclusion presupposes access to the internet, adequate digital skills, a positive attitude toward online services [15,16], and a reasonable degree of prison employee trust in incarcerated people [17]. However, during imprisonment, access to digital health care and social welfare services is limited, and the use of electronic services is characterized by a lack of trust, the use of control, and a culture that emphasizes formal security [18]. Furthermore, incarcerated people often lack digital skills and display attitudinal problems associated with the use of online services [14,19], although they are a heterogeneous group in their skills [19] and internet skills represent a multidimensional concept [20]. Gaps in digital competence can be related to not only actual skills, but also a person’s lack of confidence in his or her ability to use online services. Seo et al [21] showed that a lack of self-efficacy poses challenges for incarcerated people to access and use digital technologies. Consequently, this study focused on the significance of self-efficacy in the use of digital health care and social welfare services among incarcerated people in Finland. In particular, the study asked how incarcerated people’s self-efficacy relates to their sense of control over the use of digital health care and social welfare services.

### Use of Digital Health Care and Social Welfare Services by Incarcerated People in Finland

Incarcerated people in Finland are recognized as having a variety of social and health problems, and their ability to work is associated with major shortcomings [22]. The socioeconomic situation of people released from prison on parole is also quite weak, and according to the Criminal Sanctions Agency [23], about half (51%) are unemployed and only 26% are engaged in work. Furthermore, while 77% have a permanent home, 9% are homeless. The labor market position of people with a history of crime is weak in Finland, and is worse than in other Nordic countries [24]. The majority of incarcerated people in Finland have substance abuse problems [22]. Furthermore, according to Tuominen [25], neurocognitive and academic deficits are frequent among male incarcerated people, and the prevalence of these disorders is higher than seen in the general population. On the other hand, a Finnish prison survey (VASORA survey, 2018-2019, n=443) has shown that incarcerated people are a heterogeneous group from the perspective of perceived health status [26]. The key predictors of perceived health seem to be life expectations, a place to work or study, a permanent home, an adequate income, a permanent relationship, and a support person. In addition, the perceived health of incarcerated people in relation to other people of the same age deteriorates significantly with age. Overall, the need for health care and social welfare services for incarcerated people in Finland can be considered as significant, and in order to address these issues, incarcerated people should have access to digital social and health services already during their imprisonment and especially during their release phase.

In recent years, the digitalization of social and health care services has been rapid, both in Finland and in other Western countries. In practice, this has meant a rise in digital bookings, digital forms, virtual appointments, various forms of online therapy, and independently accessible databases and self-care programs. An important reform for the digitalization of Finnish social and health services has been the introduction of the *Kanta* service. The service includes *My Kanta* pages, where citizens can browse their own medical records and prescriptions, and reorder prescriptions online [27]. Especially with the COVID-19 pandemic, public mental health services and substance abuse treatment have also started to be provided virtually in Finland. In Finland, as part of the digital Health Village developed by university hospitals, *MentalHub* provides reliable information on mental health issues and services, tests, advice for self-care, and a variety of guidelines [28]. In addition to applying for electronic benefits, the various self-help programs and tests that are available and support substance abuse and mental health rehabilitation can be considered as important resources for incarcerated people.

In Finland, incarcerated people’s access to the internet is regulated by the Imprisonment Act. According to the Act (Chapter 12, Section 9a [29]), prisoners may be authorized to use the internet for reasons of subsistence, work, education, justice, social affairs, and housing, or for other similarly important reasons. In a closed prison, the granting of a permit requires that the prisoner’s access to nonlicensed websites is properly restricted. In the context of a prison, it is also required that any internet use does not pose a risk to the order or security of the prison, or to the safety of the prisoner or other persons.

In Finland, efforts have been made to facilitate incarcerated people’s access to the internet and digital services through the Finnish Criminal Sanctions Agency’s Smart Prison project, which follows the perspectives and goals that have emerged from discussions related to international smart corrections. The project aim is to develop the digitalization of activities and rehabilitation, and to adopt smart prison solutions in all prisons [30,31]. Currently, the development of digital services for prisons has focused specifically on the Hämeenlinna women’s smart prison, which opened in autumn 2020, where functions and facilities have been designed with technology, in order to support incarcerated people’s integration into society and create a learning environment for a crime-free life. All incarcerated women have their own terminal in their prison cell, through which they can apply for a job or housing, participate in rehabilitation or remote education, and keep in touch with relatives or other officials through white-listed sites. The cell terminals were introduced in the spring of 2021, just after the data were collected for this study.

### Self-efficacy as a Factor in the Use of Online Services

Research shows that incarcerated people tend to have deficient digital skills. This study addresses the digital self-efficacy of incarcerated people rather than their individual skills. Such a perspective considers that in addition to concrete skills, the incarcerated person’s own experience of his or her ability is an important factor. According to Bandura [32], self-efficacy is the belief “in one’s capabilities to organize and execute the courses of action required to produce given attainments.” Self-efficacy is a key factor in human agency, and a considerable amount of research has been carried out on the effects of self-efficacy in areas such as motivation, health behavior, and learning.

The importance of self-efficacy has also been extensively studied among both incarcerated people and released people. Liem and Garcin [33] argued that self-efficacy appears to be a key element in postrelease success. Correspondingly, Forste et al [34] found that specific plans and perceptions of control and self-efficacy were all associated with intentions to stay out of trouble. According to Roth et al [35], academic self-efficacy is an important predictor for participation in prison education. There is also a discussion on criminal self-efficacy that refers to a person’s perception of how capable they have been in successfully committing crimes [36]. Bailey and Ngwenyama [37] in turn emphasized the importance of self-efficacy when implementing community-based technological interventions among marginalized groups. The study by Allred et al [38] on courses that included college students (from outside the prison) and incarcerated people learning together in a prison classroom revealed the multidimensional nature of agency. Notably, they suggested that in addition to personal agency, we should look at the contextual elements of human agency. This highlights the importance of social support and cooperation in agency, but a lack of access to support can further aggravate the digital divide and increase digital exclusion [39].

A distinction can be made between general self-efficacy and specific self-efficacy such as computer or internet self-efficacy [40,41]. Internet self-efficacy has been shown to be a major factor in internet use [40], and perceived self-efficacy in computer use has been shown to have a significant positive influence on control beliefs [42]. However, the concept is not entirely unambiguous. According to Hsu and Chiu [41], general internet self-efficacy refers to “an individual’s judgment of efficacy across multiple internet application domains,” whereas web-specific self-efficacy relates to the use of a specific internet application or service. Their study shows that general internet self-efficacy influences the intention to use a digital service in the future through web-specific internet self-efficacy. Based on this previous research, it is important to distinguish between different forms of self-efficacy when looking at the use of digital health care and social welfare services among incarcerated people. In addition to general self-efficacy and internet self-efficacy, we need to analyze the specific sense of control associated with the use of digital health care and social welfare services. In accordance with the theory of planned behavior by Ajzen [43], we used the concept of perceived behavioral control, which, in this context, means the perception of the availability of skills, resources, and opportunities needed for using the technology concerned [44].

Previous studies have also found links between demographic factors, such as gender, age, and education level, and internet skills [20,45]. However, according to Noujaim et al [46], demographics and incarceration-related factors have very little effect on the health-related self-efficacy of incarcerated people. Likewise, Loeb et al [47] found that education and years of incarceration are not significantly related to self-efficacy. According to Chu [48], family support influences internet self-efficacy among older adults, and thus, it is reasonable to assume that it is also a significant factor in self-efficacy. Based on the studies described above, it is justified to analyze not only the effects of general self-efficacy and internet self-efficacy in the sense of control associated with the use of digital services, but also the effects of background factors such as age, education level, marital status, and number of convictions.

### Aim and Hypotheses

This study examined self-efficacy and the sense of control over the use of digital health care and social welfare services among incarcerated people in Finland. The study asked the following question: How does incarcerated people’s self-efficacy relate to their sense of control over the use of digital health care and social welfare services? In addition, the study investigated who incarcerated people receive help from when they encounter problems using digital health care and social welfare services.

Based on previous studies, we formulated the following 2 hypotheses: (1) Incarcerated people’s sense of control over the use of digital health care and social welfare services depends on their general self-efficacy and internet self-efficacy and (2) Incarcerated people’s internet self-efficacy depends on their general self-efficacy.

## Methods

### Sample

A cross-sectional study was conducted using a questionnaire. The study sought to attain regional coverage in Finland, and involved 11 of Finland’s 26 prisons. The sample included 6 closed prisons and 5 open prisons. One of the closed prisons also had an open ward. In order to reach female prisoners, a closed women’s prison and an open women’s ward were included in the sample. A total of 225 people responded to the survey, and the response rate was 19.9% (225/1131).

### Measures

#### General and Internet Self-efficacy

General self-efficacy was studied using the scale developed by Schwarzer and Jerusalem [49] that included 10 items. On the scale, the possible responses to the questions are as follows: (1) not at all true, (2) hardly true, (3) moderately true, and (4) exactly true. Internet self-efficacy was studied using 8 Likert-type response questions drawn from the study by Eastin and LaRose [40]. The questions concern the use of internet hardware and software on a general level, and thus, they act as a specific measure of general internet self-efficacy. In this study, the response options for these items ranged from 1 (totally disagree) to 5 (totally agree).

#### Perceived Control

Five questions were prepared according to the concept of control by Ajzen [43]. These questions [50] were connected to the ease of use of digital services in general, perceived control of the use of digital health care and social welfare services, and confidence in learning to use new digital services. These questions were formed as statements and were evaluated on a Likert-type scale ranging from 1 (totally disagree) to 5 (totally agree).

#### Help With Digital Services

Respondents were also asked who they had received help from when they had problems using digital health care and social welfare services. In particular, the study examined how many of the respondents received help from friends, family members, prison employees, health care and social welfare employees, nongovernmental organizations (NGOs), religious organizations, and volunteers.

#### Sociodemographic Characteristics

The respondent’s age was examined, as we can assume that a person’s age has a negative association with digital technology use. Education level was measured using the following answer options: no primary school, primary school, secondary education, and higher education. The respondents were asked about their gender and marital status. In addition, their number of convictions, possession of bank IDs, and use of various health care and social welfare services during the past year were asked as background questions.

### Procedure

The questionnaire used in the study was pretested using former incarcerated people (the training of experts by experience) as respondents (n=11). They were also asked questions related to the structure of the questionnaire, the ease of answering questions, the comprehensibility of the questions, and the clarity of the presented answer options. Based on the testing, minor changes were made to the layout of the form, but no changes were made to the questions themselves.

The data collection took place using a paper questionnaire between November 2020 and January 2021. The practice of data collection was agreed upon separately with each prison director. Data collection was carried out by a project worker (2 wards), university students working on the project (3 prisons), and prison staff. Responses were returned anonymously using sealed envelopes.

### Statistical Analysis

The data were analyzed using IBM SPSS Statistics for Windows (version 27; IBM Corp). Averaged composite variables of individual questions were created, and their internal consistency was estimated using Cronbach α. The normality of distributions was checked using histograms, and they showed the distributions of composite variables as being close to normal.

The actual statistical analyses were conducted using the Pearson product-moment correlation coefficient, 2-tailed *t* test, and linear regression analysis with the Enter method. Before carrying out regression analyses, the validity of the conditions was checked. The normality of the residual distributions and the linearity condition were checked graphically, and the multicollinearity between the independent variables was examined using variance inflation factor coefficients. For regression analysis, dummy variables of marital status (1=married or common-law marriage; 0=unmarried or divorced) and education level (1=secondary or higher education; 0=no secondary education) were generated.

The indirect effects of general self-efficacy and the interaction between general self-efficacy and internet self-efficacy were analyzed using the Hayes bootstrapping method. Mediation and moderation analysis was carried out using PROCESS macro 3.5.3 for SPSS, and 5000 bootstrap samples were used. The indirect effect was considered significant when the upper and lower bounds (95% CIs) did not contain a value of zero.

### Ethical Considerations

The study was conducted according to the ethical principles of the Finnish National Board on Research Integrity [51] and the WMA (World Medical Association) Declaration of Helsinki [52]. Ethical approval for the study was received from The Human Sciences Ethics Committee of the Helsinki Region Universities of Applied Sciences (decision 6/2020; September 25, 2020). Research approval was granted by the Criminal Sanction Agency (decision 30/332/2020; October 28, 2020). Informed consent was obtained from all incarcerated people who were involved in the study.

## Results

### Respondents

The average age of the respondents was 37.8 years (SD 11.7 years), and 8.9% (20/225) of the respondents were female. The representativeness of the survey turned out to be quite good. However, in some respects, the material was not fully representative. In particular, the level of education of the respondents was quite high, and the material included an overrepresentation of incarcerated people over the age of 50 years, as well as multiple recidivists with 10 or more convictions. Correspondingly, incarcerated people aged 30-39 years and those incarcerated for the first time were underrepresented. Open prisons were also overrepresented in the data (Table 1).

**Table 1:** Background data for respondents and the overall Finnish prison system.

Characteristic			Respondents (N=225), n (%)	Overall Finnish prison system, %
**Sociodemographic factors**				
	**Gender^a^**			
		Male	205 (91.1)	91.6
		Female	20 (8.9)	8.4
	**Age^b^**			
		Under 30 years	58 (30.0)	29.7
		30-39 years	53 (27.5)	35.0
		40-49 years	45 (23.3)	20.7
		50 years and over	37 (19.2)	14.6
	**Education level^c^**			
		No basic education	6 (3.1)	5.5
		Basic education	78 (40.6)	55.5
		Secondary education	86 (44.8)	35.4
		Higher education	22 (11.5)	3.6
	**Marital status^d^**			
		Married	27 (12.7)	16.9
		Common-law marriage	43 (20.3)	22.9
		Divorced	54 (25.5)	19.9
		Unmarried	88 (41.5)	37.0
**Factors related to convictions**				
	**Number of convictions^b^**			
		1	59 (33.3)	42.0
		2	29 (16.4)	13.6
		3-4	32 (18.1)	14.7
		5-9	30 (16.9)	19.0
		10 or over	26 (14.7)	10.7
	**Prison^a^**			
		Open (prison or ward)	117 (52.0)	35.6
		Closed	108 (48.0)	64.4
	**Criminal sanctions region^a^**			
		Eastern and northern Finland	82 (36.4)	31.6
		Southern Finland	83 (36.9)	35.4
		Western Finland	60 (26.7)	33.0

^a^Reference value: All Finnish prisoners [23].

^b^Reference value: All Finnish sentenced prisoners [23].

^c^Reference value: Finnish parolees [23].

^d^Reference value: Data from the study on health, working capacity, and need for treatment of criminal sanction clients [22].

### Use of Health Care and Social Welfare Services and Received Support

A total of 74.8% (166/222) of respondents answered that they use digital applications at least a few times a year. A large proportion (128/222, 57.7%) of respondents also said they searched for health information online, while a smaller proportion reported using self-care programs (87/225, 38.7%) or self-assessment tests (74/221, 33.5%) at least a few times a year. Among the respondents, 10.8% (24/223) said that they do not use digital services.

Respondents said they received support for the use of digital health care and social welfare services, especially from friends (117/223, 52.5%) and family members (99/223, 44.4%). Among the respondents, 23.3% (52/223) had received help from prison employees, but only 13.5% (30/223) had received help from health care and social welfare employees and 5.4% (12/223) had received help from NGOs, religious organizations, or volunteers.

### Composite Variables

Three averaged composite variables were constructed, and their reliability was seen as very high (α>.9) (Table 2).

The analysis of correlations (Table 3) showed that a person’s perceived control over the use of digital health care and social welfare services, self-efficacy, and internet self-efficacy correlated significantly with each other. In particular, the dependence between control and internet self-efficacy was strong (*r*=0.708; *P*<.001). Age was negatively correlated with all of these 3 sum variables. In particular, a higher age seemed to be associated with lower internet self-efficacy (*r*=−0.421; *P*<.001) and poor perceived control over the use of digital health care and social welfare services (*r*=−0.261; *P*<.001). The number of convictions correlated negatively with internet self-efficacy (*r*=−0.293; *P*<.001) and perceived control (*r*=−0.182; *P*=.02), and as expected, the number of convictions also increased with age (*r*=0.414; *P*<.001).

**Table 2:** Data for composite variables.

Variable	Items, n	Respondents, n	Minimum value	Maximum value	Mean (SD)	Cronbach α
Self-efficacy	10	215	1.00	4.00	3.06 (0.61)	.932
Internet self-efficacy	8	218	1.00	5.00	3.77 (1.06)	.957
Perceived control	5	221	1.00	5.00	3.69 (1.04)	.908

**Table 3:** Pearson correlation analysis among the study variables.

Variable			Self-efficacy		Internet self-efficacy		Perceived control		Number of convictions		Age
**Self-efficacy**											
	*r*	1		0.346		0.360		−0.023		−0.147	
	*P* value	—^a^		<.001		<.001		.77		.045	
	n	215		210		213		173		186	
**Internet self-efficacy**											
	*r*	0.346		1		0.708		−0.293		−0.421	
	*P* value	<.001		—		<.001		<.001		<.001	
	n	210		218		214		173		188	
**Perceived control**											
	*r*	0.360		0.708		1		−0.182		−0.261	
	*P* value	<.001		<.001		—		.02		<.001	
	n	213		214		221		175		191	
**Number of convictions**											
	*r*	−0.023		−0.293		−0.182		1		0.414	
	*P* value	.77		<.001		.02		—		<.001	
	n	173		173		175		177		161	
**Age**											
	*r*	−0.147		−0.421		−0.261		0.414		1	
	*P* value	.045		<.001		<.001		<.001		—	
	n	186		188		191		161		193	

^a^Not applicable.

Incarcerated people serving their sentences in a closed prison or an open prison did not differ in terms of their self-efficacy (*t*_213_=0.155; *P*=.88), internet self-efficacy (*t*_216_=1.374; *P*=.17), or sense of control over digital health care and social welfare services (*t*_219_=0.634; *P*=.53). In the data, no significant dependence was found between education level (secondary education or no secondary education) and self-efficacy (*t*_181_=0.579; *P*=.56), internet self-efficacy (*t*_185_=−0.012; *P*=.99), or control over the use of digital health care and social welfare services (*t*_187_=1.527; *P*=.13).

### Self-efficacy and Internet Self-efficacy as Predictors of Perceived Control

Respondents’ perceived control over the use of digital health care and social welfare services was examined using 5 questions. A total of 63.2% (141/223) of the respondents considered it easy to use digital services. Just over half (117/222, 52.7%) of the respondents believed that the use of various electronic social and health care services was completely under their control. The majority of respondents (155/222, 69.8%) were confident in their ability to easily learn how to use new digital services. Among the respondents, 71.7% (160/223) totally or partially agreed with the statement “I am able to apply for various social benefits electronically (eg, unemployment assistance, labor market assistance, sickness assistance, subsistence assistance, and housing assistance).” In contrast, less than half (104/223, 46.6%) totally or partially agreed with the statement “I am able to use various digital self-care programs.”

A linear regression analysis was employed to examine the factors that explain perceived control over the use of digital health care and social welfare services. According to hypothesis 1, incarcerated people’s sense of control over the use of digital health care and social welfare services depends on their general self-efficacy and internet self-efficacy. Thus, self-efficacy and internet self-efficacy were considered as independent variables. In addition, relevant background variables, such as age, level of education, marital status, and number of convictions, were included in the regression models. The first regression model (shown in Table 4) included all of the variables that were examined, and in the second model, the nonaffected variables (*P*>.05) were removed one at a time. Both models explained perceived control well (model 1: *R*^2^=0.465; model 2: *R^2^*=0.519). The analysis showed that internet self-efficacy offered the strongest explanation of perceived control, which was also consistent with the examination of correlates. In addition, according to model 2, general self-efficacy seemed to be positively associated with perceived control. Moderation analysis using the bootstrap method showed that the effect of the interaction term (general self-efficacy × internet self-efficacy) on the sense of control was not significant (coefficient=−0.032, 95% CI −0.156 to 0.092; *P*=.61).

Next, we examined what factors explained internet self-efficacy. In particular, we considered hypothesis 2, which states the following: Incarcerated people’s internet self-efficacy depends on their general self-efficacy. In the regression analysis, self-efficacy was considered as an independent variable, and relevant background variables (age, level of education, marital status, and number of convictions) were also included in the first model (Table 5). In the second model, the nonaffected variables (*P*>.05) were removed one at a time. According to the analysis, general self-efficacy was associated with internet self-efficacy. In addition, age and number of convictions reduced internet self-efficacy. In contrast, level of education or marital status did not appear to affect internet self-efficacy or perceived control.

Mediation analysis revealed that general self-efficacy had both a direct effect and an indirect effect on perceived control over the use of digital health care and social welfare services, and internet self-efficacy was the mediating variable. According to bootstrapping analysis, the indirect effect coefficient was 0.391 (95% CI 0.202-0.577). General self-efficacy also had a significant direct effect on perceived control (indirect effect coefficient=0.253, 95% CI 0.076-0.430), but the indirect effect was stronger.

**Table 4:** Linear regression analysis with perceived control as the dependent variable.

Model			B^a^		SE^b^		β^c^		*t* (*df*)		*P* value
**Model 1^d^**											
	Constant	0.920		0.529		N/A^e^		1.741 (128)		.08	
	Self-efficacy	0.179		0.119		0.101		1.495 (128)		.14	
	Internet self-efficacy	0.611		0.071		0.634		8.637 (128)		<.001	
	Age	0.003		0.007		0.034		0.419 (128)		.68	
	Number of convictions	−0.010		0.015		−0.045		−0.625 (128)		.53	
	Education level^f^	−0.239		0.139		−0.120		−1.723 (128)		.09	
	Marital status^f^	−0.075		0.146		−0.035		−0.516 (128)		.61	
**Model 2^g^**											
	Constant	0.396		0.284		N/A		1.396 (205)		.16	
	Self-efficacy	0.253		0.090		0.145		2.814 (205)		.005	
	Internet self-efficacy	0.661		0.052		0.658		12.765 (205)		<.001	

^a^Unstandardized regression coefficient.

^b^SE: standard error.

^c^Standardized regression coefficient.

^d^*R^2^*=0.465; adjusted *R^2^*=0.440; *F*_6,128_=18.5, N=135; *P*<.001.

^e^N/A: not applicable.

^f^Dummy.

^g^*R^2^*=0.519; adjusted *R^2^*=0.514; *F*_2,205_=110.6, N=208; *P*<.001.

**Table 5:** Linear regression analysis with internet self-efficacy as the dependent variable.

Model			B^a^		SE^b^		β^c^		*t* (*df*)		*P* value
**Model 1^d^**											
	Constant	3.916		0.558		N/A^e^		7.019 (130)		<.001	
	Self-efficacy	0.361		0.145		0.196		2.493 (130)		.01	
	Age	−0.033		0.008		−0.364		−4.097 (130)		<.001	
	Number of convictions	−0.032		0.019		−0.144		−1.728 (130)		.09	
	Education level^f^	0.123		0.169		0.059		0.729 (130)		.47	
	Marital status^f^	0.091		0.180		0.040		0.504 (130)		.62	
**Model 2^g^**											
	Constant	3.408		0.495		N/A		6.879 (152)		<.001	
	Self-efficacy	0.490		0.125		0.279		3.938 (152)		<.001	
	Age	−0.027		0.007		−0.295		3.851 (152)		<.001	
	Number of convictions	−0.035		0.017		−0.159		2.100 (152)		.04	

^a^Unstandardized regression coefficient.

^b^SE: standard error.

^c^Standardized regression coefficient.

^d^*R^2^*=0.232; adjusted *R^2^*=0.203; *F*_5,130_=7.86, N=136; *P*<.001.

^e^N/A: not applicable.

^f^Dummy.

^g^*R^2^*=0.251; adjusted *R^2^*=0.237; *F*_3,152_=17.0, N=156; *P*<.001.

## Discussion

### Principal Findings

Digital health care and social welfare services play important roles in the planned release from prison and integration into society. However, in prisons, the use of the internet and digital services is limited, and many psychological factors create barriers for their use. This study highlighted the importance of self-efficacy from the perspective of digital service use. In addition to the specific self-efficacy associated with the use of the internet, general self-efficacy also has a significant effect on the sense of control over using digital health care and social welfare services.

The first hypothesis that “incarcerated people’s sense of control over the use of digital health care and social welfare services depends on their general self-efficacy and internet self-efficacy” is supported. The second hypothesis that “incarcerated people’s internet self-efficacy depends on their general self-efficacy” is also supported. The study further found that older incarcerated people and multiple recidivists see their skills in using digital services as poor.

### Reflection on the Results

In terms of the use of digital systems, the sense of control associated with such use is central [44], and depends strongly in turn on internet self-efficacy. Thus, when supporting the use of digital health care and social welfare services, general information technology skills should be taken into account in order to strengthen the individual’s internet self-efficacy. Particularly, it is not enough to only support incarcerated people in their use of individual services, but it is also essential that people learn to trust their ability to operate online. Furthermore, Reisdorf and Rikard [7] pointed out that appropriate digital skills training during re-entry could mitigate some of the adverse effects of digital exclusion during incarceration and thus facilitate the re-entry of incarcerated people.

While this study did not directly focus on the process of desistance of a person released from prison, it highlighted the importance of digital services for the reintegration of released people and their perceived control over the use of digital services during their imprisonment. Previous studies have shown that incarcerated people should have access to digital services during their imprisonment, in order to facilitate their integration into society [7,9]. In particular, research has shown that both offline and online rehabilitation measures should be implemented during imprisonment and in the re-entry phase [7]. The digitalization of services and facilities makes it possible for incarcerated people to use digital health care and social welfare services from prison, to participate in distance learning and rehabilitation, and to communicate with family or different authorities, and it facilitates their search for employment [13,14].

When supporting incarcerated people in the use of digital services, the importance of general self-efficacy should also be identified. Persons with poor general life management skills often lack the skills to use digital health care and social welfare services. For this marginalized group of people, it is not enough to pay attention to only the use of the internet or digital health care and social welfare services, and one needs to pay attention to life management skills in general. This means not only facilitating support for substance abuse and mental health problems, but also introducing a range of support measures for use in everyday life [19].

Seo et al [21] pointed out that a lack of self-efficacy poses challenges for incarcerated people in accessing and using digital technologies. Our results are consistent with this, and the results show that without adequate self-efficacy and internet self-efficacy, released people are at risk of digital exclusion. Digital exclusion and social exclusion are furthermore mutually reinforcing processes that can, however, be moderated by appropriate digital skills, technology-positive attitudes, and good access to digital services [15,16].

According to previous studies, incarceration-related factors [46] or the number of years of incarceration [47] have no significant effect on health-related self-efficacy. Consistent with this, this study found no link between the number of convictions and self-efficacy, and in contrast, the number of convictions was negatively correlated with internet self-efficacy. The perceived digital ability of multiple recidivists appeared to be lower than that of other incarcerated people. This is particularly challenging because they also have a high risk of recidivism [53]. Therefore, in the prevention of recidivism, special attention should be paid to strengthening the digital skills and internet self-efficacy of recidivists.

Previous studies have also found links between demographic factors and digital skills [20,45], but the findings have been contradictory [46,47]. The importance of sociodemographic factors, such as education level and marital status, appeared to be surprisingly low in the data. Overall, a feeling of self-efficacy was more essential than an individual’s sociodemographic factors. However, older prisoners had a low level of internet self-efficacy and thus needed special support for accessing the internet and digital services.

In general, respondents estimated that they have good control over their use of digital health care and social welfare services. In particular, making electronic applications was considered to be easy. However, respondents felt that their skills relating to self-care programs were weak, even though there were many opportunities associated with self-care programs. For example, substance abuse treatment services in prisons currently reach only some of those who need them, and digital self-care programs can significantly increase treatment coverage. Accordingly, special attention should be paid to the ease of use of self-care programs and teaching their use in the future.

Previous studies have emphasized the importance of support from employees [19] and close ones [39,45,48] for digital use by incarcerated people. According to this study, the importance of informal support from family and friends for the use of digital health care and social welfare services was significant. Several respondents reported receiving support from prison employees for their use of digital services, but there is still much room for improvement. Unexpectedly, the role of NGOs as providers of digital support seemed to be quite small among the respondents of this study.

### Limitations

The data for this study were collected from different prisons across Finland, and the representativeness of the data can be considered to be quite good. In addition, the reliability of all studied variables was high. However, there were some limitations related to the research design and data. First, since this was a cross-sectional study, the analysis showed the statistical relationships between the variables, but not the causal relationships. Second, less than 10% of incarcerated people in Finland participated in the study, and thus, the results cannot be directly generalized. As a third consideration, there were some differences in background variables between the respondents in this study and the overall prison population in Finland. Specifically, the material overrepresented people serving their sentences in an open prison, and the level of education of the respondents was also quite high. On the other hand, based on our analysis, the effect of these factors appears to be reasonably small in terms of perceived control and internet self-efficacy. However, the question of the challenges of using digital services faced by the least educated incarcerated people (ie, those with no basic education) was excluded from this research design.

As a fourth area of consideration, the findings relate to the perceived control over digital health care and social welfare services, but not to actual digital skills or the actual use of digital services. Furthermore, the study examined the self-efficacy and internet self-efficacy of incarcerated people through self-assessments, and as respondents were asked to answer questions from the perspective of their current situation (ie, the situation during their time in prison), the study does not reveal how self-efficacy or internet self-efficacy changes after release.

### Conclusions

The digitalization of prisons offers many opportunities in terms of improving access to health care and social welfare services, and increasing the efficiency of prison processes. On the other hand, the technology chosen to bring about such improvements should also address the diverse challenges of the prison environment and recognize the various risk factors that present in this unique environment. Special consideration should also be given to how digitization and development work responds to the needs of incarcerated people for their integration into society and the prevention of recidivism. In order for incarcerated people to have an opportunity to adapt to life in a digital society, access to the internet and to digital health care and social welfare services must be made available during their imprisonment. In addition, during imprisonment, attention should be paid to strengthening incarcerated people’s digital skills, with support provided by prison employees. Older prisoners and multiple returnees, in particular, tend to be excluded from the digital society. The results of this study recommend paying attention to not only developing digital skills, but also strengthening the general self-efficacy of incarcerated people. Inherent to this is the key role played by social support, training, and learning of various skills needed to pursue an everyday crime-free life.

## Abbreviations

NGO: nongovernmental organization
